# Strengthening governance for antimicrobial resistance: a One Health perspective

**DOI:** 10.20506/rst.44.3675

**Published:** 2025

**Authors:** C. Clifford Astbury, J. Singh, S.G. Naro, G. Boily-Larouche, M.J.P. Poirier, F. Emdin, A. Ruckert

**Affiliations:** 1 AMR Policy Accelerator, Global Strategy Lab, York University, 4700 Keele Street, Toronto, ON M3J 1P3, Canada; 2 School of Global Health, York University, 4700 Keele Street, Toronto, ON M3J 1P3, Canada; 3 Institute of Health Policy, Management and Evaluation, University of Toronto, Toronto, ON M5T 3M6, Canada

**Keywords:** Antimicrobial resistance, Context specificity, Governance, Multisectoral coordination, National action plan, One Health, Coordination multisectorielle, Gouvernance, Plan d’action national, Résistance aux antimicrobiens, Spécificité, contextuelle, Une seule santé, Coordinación multisectorial, Especificidad del contexto, Gobernanza, Plan de acción nacional, Resistencia a los antimicrobianos, Una sola salud

## Abstract

Antimicrobial resistance (AMR) presents a complex global health challenge requiring coordinated action across human, animal, plant and environmental sectors. Context-specific One Health governance is essential to unlocking the full potential of applying a One Health approach to effectively mitigate AMR. Drawing on empirical examples from countries implementing One Health governance, this article explores how One Health governance – defined as the structures, processes and mechanisms that enable intersectoral coordination, collaboration and decision-making across the human, animal, plant and environmental health sectors – can be used to overcome key barriers to implementing One Health approaches. The authors propose a set of normative principles, including transparency, sustainability, inclusivity and equity, to guide One Health governance, and identify six core dimensions – participation, leadership, decision-making, coordination, resourcing and accountability – that should be considered when designing governance models. The need for context-specific adaptation is emphasised, recognising that governance solutions must align with national political, institutional and socio-economic realities. While One Health governance offers considerable promise, the authors also reflect on enduring challenges, including entrenched sectoral silos, unequal power dynamics, and reliance on external funding. Sustained investment in interdisciplinary capacity strengthening, long-term resourcing strategies, and inclusive and transparent decision-making mechanisms are all needed to support resilient and effective governance in addressing AMR.

## Résumé

La résistance aux antimicrobiens (RAM) représente un enjeu sanitaire complexe et d’envergure mondiale qui requiert une action coordonnée de la part des secteurs de la santé humaine, animale, végétale et environnementale. La mise en place d’une gouvernance « Une seule santé » sensible aux spécificités de chaque contexte est indispensable si l’on veut exploiter tout le potentiel de l’approche « Une seule santé » à l’appui de mesures efficaces d’atténuation de la RAM. À partir d’exemples concrets de pays ayant mis en œuvre une gouvernance « Une seule santé », cet article examine l’apport de cette forme de gouvernance – entendue comme la somme des structures, processus et mécanismes mis au service de la coordination, la collaboration et la prise de décision concertées entre les secteurs de la santé humaine, animale, végétale et environnementale – pour lever les obstacles majeurs qui freinent la mise en œuvre des approches « Une seule santé ». À titre d’orientations de la gouvernance « Une seule santé », les auteurs proposent un ensemble de principes normatifs comme la transparence, la durabilité, l’inclusivité et l’équité, et définissent six dimensions clés à prendre en compte lors de la conception d’un modèle de gouvernance, à savoir la participation, le leadership, la prise de décision, la coordination, la mobilisation des ressources et la redevabilité. L’adaptation au contexte est une nécessité, les solutions de gouvernance devant s’aligner sur les réalités politiques, institutionnelles et socio-économiques de chaque pays. Si la gouvernance « Une seule santé » se révèle effectivement prometteuse, il subsiste d’importantes difficultés que les auteurs analysent de près, notamment le caractère profondément ancré des cloisonnements sectoriels, les dynamiques de pouvoir inégales et la dépendance aux financements externes. Un investissement soutenu dans des activités interdisciplinaires de renforcement des capacités, l’adoption de stratégies de financement durables et la mise au point de mécanismes décisionnels inclusifs et transparents sont autant de conditions nécessaires pour une gouvernance résiliente et efficace de la lutte contre la RAM.

## Resumen

La resistencia a los antimicrobianos (RAM) representa un desafío sanitario complejo y mundial que requiere una acción coordinada entre los sectores humano, animal, vegetal y medioambiental. La gobernanza del enfoque «Una sola salud», específica para cada contexto, es esencial para aprovechar al máximo el potencial de la implementación de un enfoque «Una sola salud» y reducir eficazmente la RAM. A partir de ejemplos empíricos de países que implementan la gobernanza de «Una sola salud», este artículo explora la manera en que la gobernanza de «Una sola salud» (que se define como las estructuras, los procesos y los mecanismos que permiten la coordinación, colaboración y toma de decisiones intersectoriales en los sectores de la salud humana, la sanidad animal, vegetal y medioambiental) puede utilizarse para superar los principales obstáculos para la implementación de las orientaciones del enfoque «Una sola salud». Los autores proponen una serie de principios normativos, como transparencia, sostenibilidad, inclusión y equidad, para guiar la gobernanza de «Una sola salud» e identifican seis dimensiones fundamentales —participación, liderazgo, toma de decisiones, coordinación, recursos y rendición de cuentas— que deben tenerse en cuenta para diseñar modelos de gobernanza. El artículo hace hincapié en la necesidad de adaptarse al contexto específico, reconociendo que las soluciones de gobernanza deben ser conformes con las realidades políticas, institucionales y socioeconómicas nacionales. Si bien la gobernanza de «Una sola salud» ofrece un gran potencial, los autores también reflexionan sobre los retos permanentes, como los silos sectoriales arraigados, las dinámicas de poder desiguales y la dependencia de la financiación externa. Para promover una gobernanza resiliente y eficaz en la lucha contra la RAM, se necesita una inversión sostenida en el refuerzo de capacidades interdisciplinarias, estrategias de recursos a largo plazo y mecanismos de toma de decisiones inclusivos y transparentes.

## Introduction

Antimicrobial resistance (AMR) is a global issue whose drivers and impacts cut across many sectors [1,2]. Inappropriate antimicrobial use in human and veterinary medicine, as well as in crop production, can drive the emergence and spread of AMR, with the environment also playing a role via the spread of AMR and antimicrobial residues in reservoirs such as water and soil [3]. AMR is forecast to lead to substantial productivity losses and healthcare costs for both humans and animals under current circumstances, with economic harms in multiple sectors amplified through the spread of resistant pathogens between populations [4]. For this reason, the One Health approach – an ‘integrated, unifying approach that aims to sustainably balance and optimize the health of people, animals and ecosystems’ [5] – has been acknowledged as an important part of effective action on AMR. The One Health approach is vital for sustainable AMR mitigation given that resistant pathogens span numerous populations and reservoirs, with investment in AMR mitigation positioned to support substantial savings [4] and collaborative efforts to implement packages of policies across multiple sectors predicted to yield greater health and economic benefits [6]. The evidence for the economic value of One Health initiatives is growing steadily, although additional studies on AMR-specific benefits are needed [7].

For governments, adopting a One Health approach poses a series of challenges, particularly given legacies of siloed ways of working. The One Health approach involves collaborative work and nuanced negotiations around jurisdictions, leadership and resourcing. In light of this, there is increasing recognition of the importance of One Health governance. One Health governance is defined here as the structures, processes and mechanisms that enable intersectoral coordination, collaboration and decision-making across the human, animal, plant and environmental health sectors to solve complex health problems [8]. The importance of One Health governance is receiving increasing recognition, including from the Quadripartite [9] and the World Organisation for Animal Health specifically [10]. Through working as part of the AMR Policy Accelerator, a think tank initiative at York University, and as part of the Global Strategy Lab, a bi-campus interdisciplinary research lab based at York University and the University of Ottawa, the authors have also seen first-hand the challenges encountered by in-country partners in strengthening One Health governance (Box 1). The purpose of One Health governance is to support more effective infection prevention, detection and response, with collaboration enabling more timely exchange of information and action and more efficient use of resources. This could improve outcomes not only for AMR, but also for emerging and endemic zoonoses and pandemic prevention and response [7].

**Figure 1:**
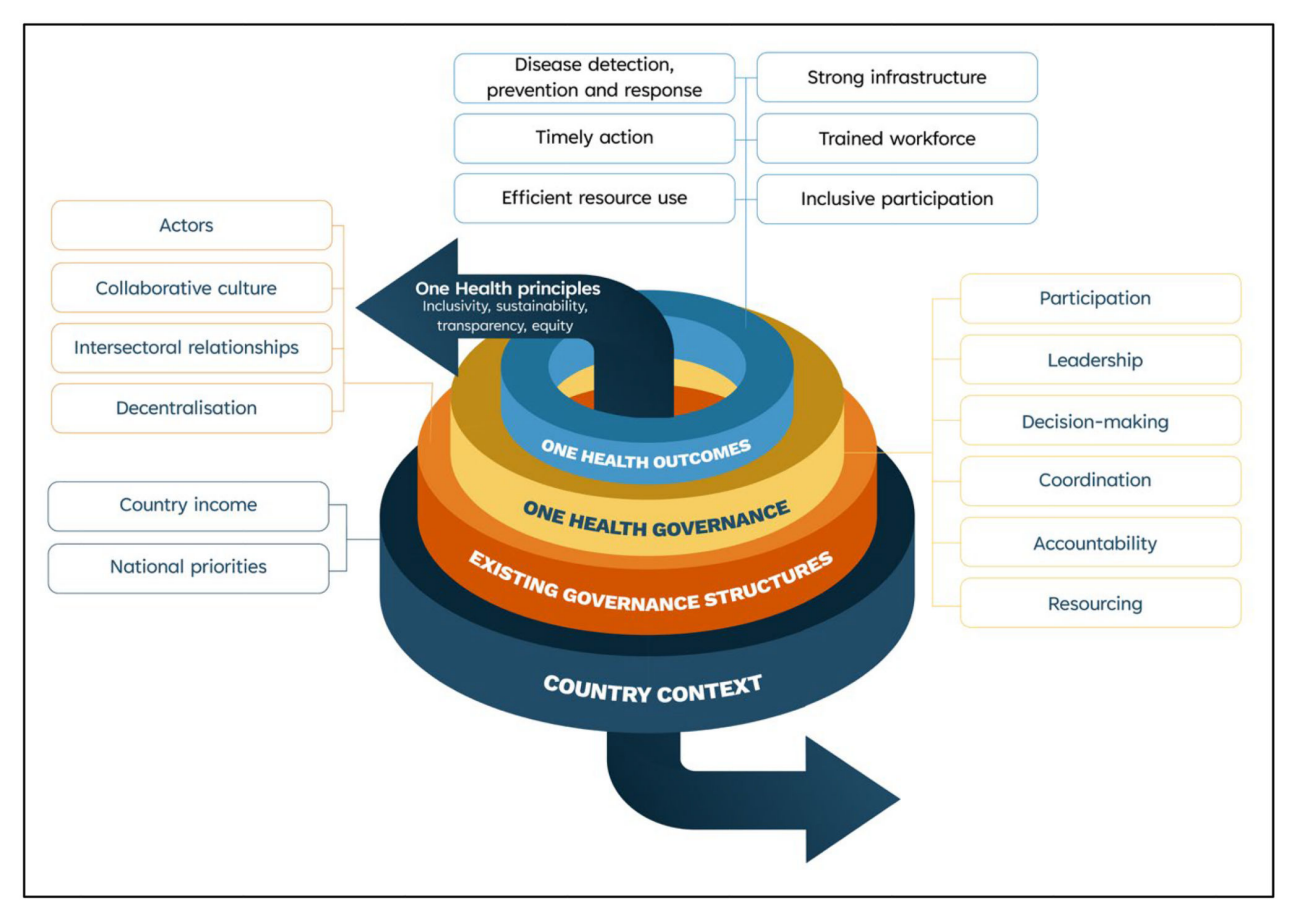
Context-appropriate One Health governance One Health governance involves six key dimensions, shaped by a country’s governance structures and context. Their interaction produces outcomes that are specific to each context. In all contexts, One Health governance should be supported by One Health principles.

Thus far, recommendations for One Health governance design and implementation have not been tailored to specific country contexts [9,11]. Different One Health governance models may be more or less feasible and effective for different countries and contexts given their specific institutional, political and economic characteristics. More specific guidance would support One Health governance implementation for countries seeking to tailor governance models to their contexts. This article outlines six dimensions of One Health governance – participation, leadership, decision-making, coordination, resourcing and accountability (Fig. 1) – that, when well-adapted to contexts and guided by normative principles, enable One Health governance to achieve its purpose. Next, common challenges encountered are identified, and finally, key recommendations on strengthening One Health governance for AMR mitigation are provided.

## Establishing a One Health ethos and related governance principles

Successful One Health governance, including for AMR mitigation, requires a One Health ethos [12] reflecting the guiding principles of inclusivity, transparency, sustainability and equity. While these principles can be considered universal and should span all contexts, context-specific One Health governance models can support these principles by effectively embedding them across different countries.

The broad-based **inclusion** of relevant stakeholders, including public sector, private sector (including partners across animal production systems) and community representatives, as well as subject-matter experts, is a crucial ingredient of the One Health approach [13]. Such inclusion creates a sense of ownership and trust amongst One Health stakeholders, which, in turn, may enhance the chances of success [14].

Inclusion, however, requires a clear understanding of the roles and responsibilities of stakeholders to enable **transparent** governance processes [13]. This entails determining specific roles for sectors and individual actors, which further deepens ownership and creates a sense of responsibility among One Health stakeholders. Establishing transparent One Health governance processes promotes fiscal responsibility and contributes to the sustainability of initiatives [15]. Transparency can also promote equitable processes, particularly in One Health contexts where stakeholders with power differentials may be involved. While transparency is a cross-cutting principle, its implementation may vary by context, for example balancing mitigating conflicts of interest with maintaining feasibility of policy processes [16].

**Sustainability** is another central ingredient in effective One Health governance, especially given that One Health collaborations are often *ad hoc*, time-bound, and resource-intensive policy initiatives. Sustainability refers to the establishment of structures and processes – such as funding stability, legislation, lasting partnerships, ongoing communications and widespread political support – that facilitate adequate resourcing to sustain initiatives over time [17,18].

Finally, **equity** is a cross-cutting principle that must drive all aspects of One Health governance. This includes supporting both equitable processes, ensuring that the perspectives and interests of different actors are integrated into policy decision-making in an equitable way, and equitable outcomes, where equitable health, economic and other outcomes are achieved through policy action and effective governance. Given the different actors and governance levels involved in One Health, power differentials can exist between sectors, but also between decision-makers and affected communities, across genders, between Global North and Global South countries, between public and private actors, or between smaller and larger enterprises [19–22]. To foster more equitable interactions, One Health governance processes must be co-designed with balanced representation, shared decision-making power, and equitable resource allocation across these axes. More equitable processes can, in turn, support more equitable outcomes, as equity-seeking groups obtain more power in decision-making processes.

## Designing One Health governance models for antimicrobial resistance

One Health governance is established within existing governance contexts, each with their own path-dependent structures, processes and mechanisms. Countries must ensure these contexts are taken into consideration when designing or adapting One Health governance (Box 2). The authors suggest six key dimensions that countries should tailor to their own context: participation, leadership, decision-making, coordination, resourcing and accountability. Across all contexts, these dimensions should be imbued with the One Health ethos described above.

**Participation** refers to the involvement of stakeholders in One Health governance, including both who participates and the role they play. The government ministries typically given formal roles focus on human health, animal health and, to a somewhat lesser extent, the environment and plant health, while other state and non-state actors may also play different roles. Designing participation involves trade-offs between inclusivity (i.e. the need for different perspectives to be represented) and timely and efficient action. Countries should reflect on which stakeholders to engage, but also on the role each stakeholder should play to facilitate inclusive governance processes. For example, Nigeria strategically engaged with distinct stakeholder groups in developing its second AMR National Action Plan (NAP) [29]. This included extensive consultation with stakeholders from multiple sectors and regions as part of an initial consultation and situational assessment, combined with the establishment of a smaller technical working group to steer NAP development. In the published NAP, this emphasis on inclusive participation continues, with multisectoral strategic, coordinating and technical bodies, as well as core principles around engaging non-state actors such as communities and the private sector [30].

**Leadership** in One Health governance must balance selecting leaders who are committed to driving the effort forward with ensuring that all sectors involved maintain a sense of ownership. This issue is challenging within an intersectoral context. Some countries have chosen to house leadership of One Health governance within a single sector, most often human health [31]. In the case of AMR, this predominance of the human health sector reflects, at least in part, the role of the World Health Organization in agenda-setting and its close connections with in-country human health stakeholders [32]. In some cases, single-sector leadership can result in a loss of engagement and sense of ownership from other sectors, as well as bias in decision-making and prioritising. Other countries have implemented either shared or rotating leadership, or have located leadership of One Health governance at a higher political level, such as a presidential or prime ministerial office [33]. However, these approaches may also have drawbacks. For example, high-level political actors may lack the bandwidth needed to drive progress on One Health issues [23]. The appropriate leadership mechanism may depend on the extent to which One Health-relevant diseases are a high-level political priority in a country: where One Health is a key priority, high-level political actors may be sufficiently engaged to act as effective One Health leaders. Thailand’s work on implementing AMR policy illustrates how these trade-offs in leadership for cross-sectoral work may be effectively navigated [34]. The national steering committee charged with implementing the country’s AMR NAP benefits from high-level political support, being appointed by the prime minister and chaired by the deputy prime minister. Veterinary Services (through the Department of Livestock Development) were also formally engaged in leadership and priority-setting within this committee, helping align animal and human health priorities [35]. However, this engagement is paired with coordination between non-political leaders at the operational level, making efforts on AMR more resilient to shifts in political priorities and leaders [34].

Processes and mechanisms to enable **shared decision-making** for public policy are an important part of One Health governance. Designing decision-making mechanisms involves decisions around who should feed into decisions (which can involve both state and non-state actors), what kind of evidence informs decision-making, and how these inputs should be weighed. In shared decision-making, more powerful actors can use formal and informal pathways to give their interests greater weight. This presents a challenge to One Health governance, as there may be power differentials between actors, and decision-making processes should be designed to support equity between actors. Overall, more structured, consultative approaches to decision-making can support both transparency and equity. Some examples of these types of approaches have already been implemented, including standardised processes for cross-sector priority-setting such as the United States Centers for Disease Control and Prevention Zoonotic Disease Prioritization workshops [36] or the National Bridging Workshops organised by the Tripartite to link the human health-focused International Health Regulations with the animal health-focused Performance of Veterinary Services [37].

National Bridging Workshops encompass a number of One Health topics, including AMR, and have been used in over 55 countries to develop prioritised ‘roadmaps’ for action that have been agreed by representatives from multiple sectors [38]. The AMR Policy Accelerator has also developed and implemented the Smart Choice Process, a decision-making tool that takes a country-led, One Health approach to prioritising actions included in countries’ AMR NAPs through structured consultation and engagement with key stakeholders from different sectors [39].

**Coordination** involves processes and mechanisms by which activities are coordinated across actors and sectors, including structures for information-sharing. Coordination mechanisms can range from less formalised (e.g. *ad hoc* working groups, meetings) to more formalised (e.g. formal mechanisms enshrined in law, memoranda of understanding). A key mechanism deployed by countries to support the coordination of One Health governance is the establishment of a multisectoral coordination mechanism. These mechanisms should be adapted to existing governance structures. For example, in countries with high levels of decentralisation, subnational One Health coordination can allow for more effective action; in cases where this is lacking, disease detection and response can be slowed down by the need to escalate information to national focal points [33,40]. Another key approach to coordinating One Health action involves legislation coordination and harmonisation [18,41]. A lack of harmonised legislation is frequently cited as a barrier to One Health governance, as laws can sometimes emerge in silos to meet sector-specific needs. An example of the One Health approach to regulation includes livestock sector restrictions on the use of medically important antimicrobials for human medicine [41]. Efforts in several countries in the European Union and Canada as well as certain areas of the United States of America have focused on restricting the use of medically important antimicrobials in livestock production, providing an example of an effort driven by the animal health sector to support antimicrobial stewardship for both human and animal populations [42].

**Resourcing** encompasses both the origin of resources for One Health governance activities and the way these resources are made available: either through dedicated financing and staffing for One Health governance, or by integrating these new activities into the obligations for existing budgets and staff. In low- and middle-income countries, One Health governance may be supported by external development partners. These external partners may be the only funding source of One Health governance or may be paired with domestic contributions [20]. External funds are a double-edged sword for One Health governance: they are often cited as key drivers in putting the One Health approach on the agenda, but they may also threaten sustainability and domestic ownership if external agencies play too strong a role in leadership and decision-making. Both development partners and national governments should prioritise long-term financial sustainability in their planning, and where possible, efforts should be jointly funded by national organisations to increase country ownership. Thailand has approached this issue by taking a pooled approach to funding the implementation of its AMR NAP, whereby national and international contributors pool their contributions, enabling synergies across funding sources and the streamlining of reporting [43]. Beyond this, countries may choose to fund and staff One Health governance activities either through dedicated resourcing or by embedding budgets into other activities. While the merits of each approach depend on context, several countries have had success in having a dedicated staff member responsible for coordinating a larger group of actors [15,44]. For example, in Singapore, the creation of a dedicated AMR Coordinating Office to support the implementation of the country’s AMR NAP has been cited as an important factor in supporting NAP implementation [15], as Office staff could dedicate their time to maintaining coordination between the different agencies involved. Finally, the private sector has provided substantial resources to support AMR mitigation, for example where the production sector has implemented industry-led measures to support antimicrobial stewardship [42], and by collaborating with other organisations to support surveillance of AMR and antimicrobial use [45].

Finally, **accountability** encompasses both structures for making actors responsible for specific actions and mechanisms for supporting adherence to these responsibilities. Outlining clear roles and responsibilities for actors involved in One Health governance is an important aspect of supporting accountability and maintaining sustainable governance mechanisms [20]. Malawi’s AMR NAP was a strong example of this, delineating clear roles for national ministries, development partners and civil society organisations, along with clear targets for planned action, which has enabled some key successes in AMR mitigation [46]. However, while designating accountable actors is a central part of promoting accountability, it does not always ensure that responsibilities are met in practice [47]. Accountability must be supported by adequate resourcing, as well as knowledge mobilisation and capacity strengthening among implementing actors, including, in the animal health sector, and with veterinarians and producers. Implementing appropriate monitoring and evaluation structures can also support accountability, as can public reporting of monitoring results. For example, in the Netherlands, results from AMR surveillance systems are published in the annual NethMap/MARAN report, which integrates data from human and animal populations and healthcare settings [48]. Overall, accountability structures must consider a country’s unique resources and capacities so that monitoring and evaluation results are positioned to inform decision-making.

## Challenges in One Health governance for antimicrobial resistance

Financial and human resources are a major constraint for One Health governance. In addition to issues around resourcing from development partners discussed above, barriers related to the availability and allocation of resources also exist. Resources for cross-sectoral coordination can be challenging to obtain, particularly in resource-constrained settings [49]. Capacity strengthening for professionals is an important aspect of this, and countries are investing in AMR-focused training for medical professionals as well as for policy-makers [50], sometimes with support from internationally funded agencies such as ReAct [51]. Explicitly One Health-oriented training has also been developed and delivered to these professional groups in the human, animal and environmental sectors [44]. Capacity strengthening can be important for establishing a shared language, the lack of which is often perceived as an important barrier for One Health approaches, as well as building understanding and enthusiasm for One Health approaches to AMR.

Breaking down sectoral silos is another critical challenge. Institutional fragmentation, different sectoral priorities, and long-standing professional cultures hinder cross-sectoral collaboration. This is exacerbated by a lack of understanding among stakeholders of the added value of the One Health approach, especially given the challenges of demonstrating this added value [52]. One Health efforts can be seen as a concern predominantly for animal health, with other sectors being less engaged [19]. This can be a particular challenge for AMR mitigation where there can be a lack of focusing events, unlike other One Health-relevant domains where rapidly spreading outbreaks may catalyse collaboration. One Health governance structures that have been developed to address, for example, emerging zoonoses do not always lead to the same level of collaboration around AMR [26]. In these contexts, several strategies have been deployed to foster engagement. Making an evidence-informed case on, for example, the economic benefits of AMR mitigation can help to engage sectors for which AMR is otherwise not a priority. As described above, appropriate leadership structures may also support the engagement of siloed actors, including high-level political leadership or shared leadership between sectors. Politically focused initiatives, such as Tanzania’s Parliamentarians Alliance for Antimicrobial Resistance [53], can support political engagement in a One Health approach for AMR. Finally, consultation processes to inform AMR NAP development and implementation, which have been carried out in a number of countries [27,29], can build a sense of shared ownership of AMR efforts and reorient attitudes to focus on shared solutions.

Lastly, the development of integrated surveillance systems is an important aim of One Health governance that presents numerous challenges. Because AMR emerges and spreads across the human, animal, plant and environmental sectors, integrated One Health surveillance is critical for capturing the full picture of transmission dynamics and identifying opportunities for coordinated AMR interventions. Effective One Health surveillance systems, such as the Canadian Integrated Program for Antimicrobial Resistance Surveillance, known as CIPARS, and the Danish Integrated Antimicrobial Resistance Monitoring and Research Programme, or DANMAP, require harmonised data collection, coordinated reporting systems and strong connections between surveillance and policy responses [54]. However, inequitable resourcing across sectors can pose challenges for surveillance, particularly as animal health surveillance systems are typically less well-established and resourced [46]. Progress has been made globally with implementing integrated surveillance systems for AMR, including the establishment of a Quadripartite Technical Working Group to issue guidelines for One Health surveillance, but the operationalisation of integrated surveillance systems in AMR remains limited, particularly in low- and middle-income contexts [54]. A stepwise approach to strengthening animal health surveillance, designing sector-specific data collection and analysis with integration in mind, will be key to expanding integrated surveillance systems globally [54].

## Key recommendations for enhancing One Health governance for antimicrobial resistance

Approaches to designing effective One Health governance models for AMR must start with a strong understanding of the institutional and resourcing context of a country. This includes being aware of existing governance structures in order to avoid duplication of efforts. A good assessment of existing capacity and infrastructure is also key to designing governance mechanisms that build on current strengths and acknowledge current limitations. This means that **initial One Health capacity assessments should start with a broad canvassing of the wider governance landscape** to identify entry points for One Health governance. This should include assessments that cover all One Health sectors, as structures and processes may vary between human health, animal health and the environment.

Strengthening One Health capacity is an important enabler of strong One Health governance. A lack of shared language is often cited as a barrier to implementing the One Health approach. **One Health capacity can be strengthened through training**, including embedding One Health into formal education (including through shared training for animal and human health professionals) and expanding access to workplace-based One Health training for policy-makers and practitioners. This will help foster shared understandings, mutual respect and positive relationships across disciplines and sectors.

Building on the foundations of strong contextual awareness and One Health capacity, countries **must tailor their One Health governance models to their specific context**. Table I presents a suite of recommendations focused on the six dimensions of governance outlined above: participation, leadership, decision-making, coordination, resourcing and accountability. Within the table, country examples that illustrate the recommended approaches are also noted. These examples are promising but would benefit from additional in-depth study to establish causal and explanatory evidence, determine policy effectiveness and develop more concrete guidance.

## Conclusions

Collaborative approaches to address cross-cutting global challenges are increasingly needed. One Health governance provides a promising way for governments to coordinate for more effective action on AMR and other One Health priorities. As One Health governance gains traction around the world, maximising effectiveness and impact requires ensuring that these efforts are embedding a One Health ethos centred around inclusivity, transparency, sustainability and equity, and are adapting to national political, cultural and socio-economic contexts. Going forward, national governments must design adaptable, context-specific One Health governance models to drive effective AMR policy implementation.

## Figures and Tables

**Table I:** Key recommendations for strengthening context-specific One Health governance

Dimensions of One Health governance	Key recommendations
**1. Ensure inclusive and effective participation**	− Map relevant state and non-state actors and assess their capacity to participate and engage in AMR NAP development *See Nigeria’s strategic engagement with different actors throughout AMR NAP development and implementation 29* − Establish flexible participation mechanisms that balance inclusivity with efficiency *See Singapore’s establishment of regular meetings of its AMR working group, allowing ongoing sharing of progress by different agencies 15*
**2. Design leadership to promote shared ownership and commitment**	− Where feasible, engage high-level political champions to anchor One Health efforts *See Zambia’s multiple governance tiers engaging both political and operational leadership to sustain momentum for its AMR NAP 55* − Where leadership is housed within sectors, use shared or rotating leadership models to avoid dominance by any single sector *See Sweden’s joint coordination of work on AMR by the Public Health Agency and the Board of Agriculture 56*
**3. Support transparent and inclusive decision-making**	− Develop structured and transparent decision-making processes and use participatory tools to include diverse perspectives from state and non-state actors *See examples of standardised tools from USAID and the Quadripartite to support inclusive cross-sectoral decision-making around One Health priorities [36,38]* − Explore and adapt existing structured decision-making models and tools to fit national contexts *See the WHO Benchmark on International Health Regulations tool, which builds on countries’ existing structures, such as multisectoral mechanisms on AMR, and coordinates with partners to leverage existing resources 57*
**4. Establish formal or informal One Health multisectoral coordination mechanisms**	− Design coordination mechanisms to align with national governance systems *See Canada’s exploration of top-down and network-based governance models for AMR NAP implementation within a federal system 27* − Consider vertical and horizontal coordination mechanisms *See the DRC’s example of establishing One Health platforms at the national level as well as the subnational level to support multilevel coordination 23* − Consider coordination as a foundational element of One Health governance, but not as its goal *See concerns among stakeholders that there are resources for coordination meetings but not for implementation of actions included in their AMR NAPs (for example in Ghana and South* *Africa [51,58])*
**5. Plan for long-term resourcing and sustainability**	− Pursue co-financing arrangements between countries and development partners to foster ownership *See Thailand’s approach to pooling resourcing from national and international funders 43* − Develop strategies to strengthen domestic financing of One Health governance *See exploration of alternative financing models for action on AMR in the African region by senior leaders at ReAct Africa, given reduced resources for global health and development 59*
**6. Build accountability through clear roles and monitoring**	− Define responsibilities clearly across sectors and institutions, as well as outlining clear mandates for coordination bodies *See Malawi’s approach to clearly delineating responsibilities for state and non-state actors within its One Health-informed AMR NAP 46* − Develop monitoring and evaluation frameworks tailored to national capacities and priorities *See Mongolia’s approach to developing baseline data that was previously unavailable, which could then be used for One Health monitoring and evaluation 60* − Use evaluation data for continuous learning and adaptation, not only for compliance *See the use of AMR surveillance data to inform China’s ban on colistin for growth promotion in livestock 61*

AMR: antimicrobial resistance DRC: Democratic Republic of the Congo NAP: national action plan USAID: United States Agency for International Development WHO: World Health Organization

## Box 1 How research, advisory and capacity-strengthening services are supporting policy on antimicrobial resistance

The AMR Policy Accelerator, which is based in Canada, provides research, advisory and capacity-strengthening services to support evidence-informed policy on antimicrobial resistance (AMR). In this context, countries have exhibited growing interest in strengthening One Health governance, as reflected by the more frequent inclusion of a One Health governance objective in national action plans, as countries identify challenges and opportunities inherent in collaborative approaches to mitigate AMR. The Accelerator’s country partners have raised some of the specific challenges they face, such as harmonising data from surveillance systems established in different sectors, and building the trust needed to integrate and share these sometimes sensitive data. The Accelerator’s work with decision-makers also highlights how governance involves both formal and informal aspects. For example, policy actors emphasise that what is written formally does not always translate to on-the-ground realities, and formal governance mechanisms need to be supported with adequate resourcing and accountability mechanisms. Finally, discussions have underscored the trade-offs decision-makers must negotiate when implementing One Health governance. For example, resourcing One Health governance efforts with existing sector-specific staff can increase efficiencies but may lead to staff members being overburdened and cross-sectoral efforts being deprioritised.

## Box 2 One Health governance across contexts

In the **Democratic Republic of the Congo (DRC)**, an executive order from the Ministry of Higher Education has established a national One Health platform to coordinate different ministries around One Health issues, including antimicrobial resistance (AMR) [23–25]. As the DRC is a relatively decentralised nation, the government is also implementing a decentralisation process for One Health governance, supporting provinces to establish subnational One Health platforms that are mandated to implement national strategy according to local context. The development of the DRC One Health national action plan (NAP) was supported by donors and development partners including the United States Agency for International Development, although the NAP recognises funding and sustainability as core concerns.

In **Vietnam**, multisectoral coordination for One Health has occurred primarily through *ad hoc* working groups formed in response to crises, such as outbreaks of high pathogenicity avian influenza, for which a regulatory framework facilitates coordination between national government departments, chiefly the Ministry of Health and the Ministry of Agriculture and Rural Development [26]. While this may be an effective mechanism for emergency response, it may be less appropriate for dealing with chronic issues such as AMR, which lack events such as outbreaks to catalyse collaboration.

In **Canada**, the federal system poses challenges to country-wide One Health governance, as subnational authorities have a relatively high level of autonomy. On the publication of its AMR NAP in 2023, the federal government charged a steering committee with broad-based, multisectoral consultation to inform the potential development of a national AMR governance mechanism [27,28]. This consultation revealed a substantial amount of often siloed AMR activities in different regions, sectors and governance levels. The process led the steering committee to recommend different governance approaches, including both a ‘top-down’, centralised approach and a network approach to governance. Meanwhile, various state and non-state actors are involved in coordination for AMR in Canada. For example, the non-profit organisation Animal Health Canada, funded by national and subnational government bodies as well as private sector organisations, coordinates across different governance levels and supports the implementation of the AMR NAP in animal health. Despite challenges related to federal governance, the Canadian Integrated Program for Antimicrobial Resistance Surveillance, or CIPARS, is recognised as one of the leading integrated surveillance systems for AMR globally.
